# Effects of chemical-based fertilizer replacement with biochar-based fertilizer on albic soil nutrient content and maize yield

**DOI:** 10.1515/biol-2022-0057

**Published:** 2022-05-18

**Authors:** Dawei Yin, Xiangyu Yang, Haize Wang, Xiaohong Guo, Shiqiang Wang, Zhihui Wang, Guohua Ding, Guang Yang, Jianing Zhang, Liang Jin, Yu Lan

**Affiliations:** College of Agricultural Science, Heilongjiang Bayi Agricultural University, Daqing 163319, China; College of Tillage and Cultivation of Heilongjiang Province, Heilongjiang Academy of Agricultural Sciences, Harbin 150000, China; College of Plant Nutrition and Resources, Beijing Academy of Agriculture and Forestry Sciences, Beijing 100097, China; Key Laboratory of Biochar and Soil Improvement, Ministry of Agriculture and Rural Affairs, P. R., Shenyang Agricultural University, Shenyang 110161, China

**Keywords:** albic soil, biochar, maize, nutrient, crop production

## Abstract

Biochar-based fertilizers are used to improve soil’s physiochemical and biological properties and increase fertilizer utilization rate. Therefore, a technological model of biochar-based fertilizers is essential for the reduced application. This study was conducted to determine the effects of the different levels of biochar-based fertilizer applications on soil and plant nutrient content, as well as maize yield. Biochar-based fertilizer increased the total N content of maize stem and kernel and the total P content of maize axis and kernel. Biochar-based fertilizer increased the total P but decreased the total K of maize plants while increasing the fertilizer’s partial productivity. Treatment B1 (600.00 kg hm^−2^ of biochar-based fertilizer) increased the dry-matter weight of the maize at silking and filling stages by 1.60 and 15.83%. Treatment B1 increased the ear length, diameter, and plant height. Compared with BCK (600.00 kg hm^−2^ of conventional fertilizer), the yield of B1 was increased by 9.23%, and the difference was significant (*p* < 0.05). Biochar-based fertilizer treatments B2–B5 (biochar-based fertilizer reduced by 5–20%) reduced maize yield, but there was no significant difference between their yield and BCK. This study aimed to provide a basic understanding and reference for maize fertilizer reduction with good application prospects.

## 1 Introduction

To meet the increasing food requirements of the growing population, China is the largest chemical fertilizer consumer in the world (i.e., synthetic nitrogen (N), phosphorus (P), and potassium (K) fertilizer use were approximately 29, 30, and 26%, respectively, of the global total for agriculture from 2002 to 2019) [1]. However, the long-term overuse of chemical fertilizers can lead to increased N and P water pollution, soil degradation, and reductions in fertilizer-use efficiency and crop yield [2]. The major factors restricting the development of China’s maize industry are superfluous fertilizer input and low utilization of fertilizer; therefore, an urgent study for a new fertilizer application model is required [3]. Throughout the world, 32 countries or regions exhibit similar distributions of albic soil, and the total area of albic soil in China is approximately 5.273 million ha [4]. Owing to the severe problems caused by its dense physical structure, poor nutrient content, and low biological activity, albic soil is characterized as low-yielding [4,5]. Therefore, improving the low-yielding albic soil is strategically important to ensure food security.

Recently, biochar production and utilization have emerged as a widely recognized research area of great concern to experts and scholars worldwide. Biochar is rich in C and possesses a large surface area and strong adsorption capacity, several micropores, and other nutrient elements [6,7]. Straw and other biomasses are prepared as biochar and applied to the soil to considerably reduce soil bulk density; increase soil porosity; improve soil temperature, microecological environment, and nutrients; stimulate and promote soil microbial reproduction; and promote growth and development of a variety of crops [8,9].

Researchers have introduced biochar-based fertilizer to increase crop production and nutrient-use efficiency (NUE) as it has demonstrated great potential to be used as a slow-release fertilizer [10]. Biochar can be used as a carrier for nutrient delivery due to its unique physical and chemical properties, and various types of nutrients have been incorporated into the matrix of biochar [11,12,13]. Previous studies have demonstrated that biochar-based fertilizer could reduce the loss of nutrients and increase the NUE by the crops in the long term compared with conventional fertilizer [14,15,16].

Biochar-based fertilizer shows many advantages as compared with conventional fertilizer [15,17]. However, a few reports exist on biochar-based fertilizer applications to replace those of chemical fertilizers in the cold region of Northeast China. Therefore, this article studies the effects of biochar-based fertilizer instead of chemical fertilizer on the nutrient content of northeast albic soil, maize nutrient absorption, maize dry matter accumulation, and maize yield, and reveals the mechanism of biochar-based fertilizer reduction on maize yield, to provide theoretical basis and technical reference in innovating the technical model of maize-reduced fertilization.

## 2 Materials and methods

### 2.1 Overview of the test area

The test area is located in the Modern Agriculture Demonstration Park (45:43:59.40 N, 132:29:59.22E) of 850 Farm, Hulin City, Heilongjiang Province, China. It belongs to the temperate humid to subhumid continental monsoon climate (dry spring, humid, June to August), with an annual average temperature of 3.5°C and an annual average rainfall of 551.5 mm.

### 2.2 Test materials

The test soil type was albic soil of northeast meadow. The background values of basic soil nutrients were 34.8 g kg^−1^ organic matter, 1.70 g kg^−1^ total N, 0.877 g kg^−1^ total P, 162 mg kg^−1^ alkali-hydrolyzable N, 45.3 g kg^−1^ available P, and 97.0 g kg^−1^ available K in 0–20 cm surface soil. The values for pH and CEC were 5.35 and 10.16 cmol kg^−1^, respectively. Biochar-based fertilizer was provided by Shenyang Longtai Bioengineering Co., Ltd, Liaoning, China (total nutrient content ≥ 45%, i.e., N + P_2_O_5_ + K_2_O ≥ 45%). The maize variety was Kenyu 6 and was provided by the Maize Center of Heilongjiang Bayi Agricultural University.

### 2.3 Experimental design

The field experiment was laid out in a randomized block design with seven treatments, each replicated three times. The treatments imposed comprised: BKB (no fertilization); BCK (600.00 kg hm^−2^ of conventional fertilizer – 46% urea 225.00 kg hm^−2^, 64% diammonium phosphate 225.00 kg hm^−2^, and 60% potassium sulfate 150.00 kg hm^−2^); B1 (600.00 kg hm^−2^ of biochar-based fertilizer – with the same quality as the BCK treatment); B2 (biochar-based fertilizer reduced by 5%, 570.00 kg hm^−2^); B3 (biochar-based fertilizer reduced by 10%, 540.00 kg hm^−2^); B4 (biochar-based fertilizer reduced by 15%, 510.00 kg hm^−2^); and B5 (biochar-based fertilizer reduced by 20%, 480.00 kg hm^−2^). Each treatment plot area was 666.67 m^2,^ and maize planting density was 52,500 plants/hm^2^, and the row spacing was 65 cm. Each treated fertilizer was used as base fertilizer, and no topdressing was required afterward. To avoid yield losses, conventional management practices were performed on the maize plants, weeds, insects, and diseases, controlled by either chemical or manual methods.

### 2.4 Sample collection

The soil sampling was carried out at the key growth stages of maize, which included the silking, grain-filling, and maturity stages. Maize rhizosphere soil with a depth of 0–20 cm was collected at the rice maturity stage using a stainless steel soil drill with a diameter of 2 cm, and 10 points were randomly selected from each treatment. After removing the roots, weeds, soil animals, and other impurities, they were mixed and used as a repeated soil sample for the same treatment. The soil sample was air-dried to analyze its chemical properties.

Ten maize plants were continuously investigated in each plot at their key growth stages to determine changes in dry matter weight. The maize plants were monitored continuously at the jointing and filling stages, and soil and plant analyzer development (SPAD) values of functional maize leaves (inverted three leaves) were measured. The yield of maize was measured at the maturity stage.

### 2.5 Soil nutrient determination

Soil pH was measured in 1:2.5 ratio soil solutions (with deionized water) using a pH meter. The soil organic matter (SOM) content was measured using the high temperature–volume method, with heating and oxidation by potassium dichromate. For total N, H_2_SO_4_ was used as an accelerator for digestion, and then the Kjeldahl analytic method was used. The soil alkali-hydrolyzable N was measured using the alkaline hydrolysis diffusion method. Available P was extracted using sodium bicarbonate and determined with ultraviolet spectrophotometry (TU-1810; Beijing Pgeneral Instrument Co. Ltd., Beijing, China). Total P was measured using the alkali fusion-molybdenum antimony anti-spectrophotometric method. Soil total K(TK) and available K (AK) were quantified using inductively coupled plasma-atomic emission spectrometry (ICPS-7500; Shimadzu, Japan). All the previously mentioned chemical indexes were measured according to Soil Agrochemical Analysis published by China Agriculture Press [18].

### 2.6 Calculations for fertilizer agronomic efficiency

Fertilizer agronomic efficiency and fertilizer partial productivity were calculated using the following formula [19]:

\begin{array}{c}\text{Fertilizer}\hspace{.25em}\text{agronomic}\hspace{.25em}\text{efficiency}\hspace{.25em}\text{(kg}\hspace{.25em}{\text{kg}}^{-1}\text{) }\\ \hspace{1em}=\text{(Yield}\hspace{.25em}\text{of}\hspace{.25em}\text{fertilized}\hspace{.25em}\text{area}-\text{yield of unfertilized area)}\\ \hspace{2em}\text{/amount of fertilizer applied,}\end{array}]

\begin{array}{c}\text{Fertilizer}\hspace{.25em}\text{partial}\hspace{.25em}\text{productivity}\hspace{.25em}\text{(kg}\hspace{.25em}{\text{kg}}^{-1})\\ \hspace{1em}=\text{yield/amount}\hspace{.25em}\text{of}\hspace{.25em}\text{fertilizer}\hspace{.25em}\text{applied}\text{.}\end{array}]

### 2.7 Measurement of dry matter weight, SPAD value, and actual yield of maize

Dry matter weight of stem, dry matter weight of sheath and leaf, dry matter weight of ear, and total dry matter weight of the abovementioned parts were determined by drying: each plant part was dried first in an oven at 105°C for 30 min and dried to constant weight at 80°C for moisture loss. SPAD values of functional maize leaves were measured using a SPAD-502 chlorophyll meter produced by Minolta Co., LTD (Tokyo, Japan). At the mature stage of maize, the area of each plot was determined to be 80m^2^, and the actual yield of maize was calculated.

### 2.8 Data analysis

SPSS 19.0 statistical software was used for variance analysis, LSD was used to test the significance of difference (*p* < 0.05), and Microsoft Excel 2010 was used for plotting.

## 3 Results

### 3.1 Effects of biochar-based fertilizer on SPAD value of maize

B1 and B2 increased the SPAD value of maize at silking stage (Table 1). The SPAD values of B1 and B2 increased by 7.56 and 1.33%, respectively, compared with that of BCK. However, all treatments reduced the SPAD value at the filling stage.

**Table 1:** Effect of biochar-based fertilizer on SPAD value of maize

Treatment	Silking stage	Filling stage
BKB	44.14 ± 2.37^ab^	42.44 ± 3.91^a^
BCK	43.66 ± 1.46^bc^	46.50 ± 0.80^a^
B1	46.96 ± 1.96^a^	41.98 ± 3.86^a^
B2	44.24 ± 2.31^ab^	41.50 ± 1.66^a^
B3	43.50 ± 3.28^bc^	42.54 ± 5.68^a^
B4	46.14 ± 1.63^ab^	44.84 ± 2.99^a^
B5	40.74 ± 1.51^c^	34.22 ± 3.15^a^

Note: Letters a–c in the same column indicate significant difference at *p* < 0.05 (*n* = 10, LSD test). BKB (no fertilization); BCK (600.00 kg hm^−2^ of conventional fertilization); BCK kg hm^−2^, 64% diammonium phosphate 225.00 kg hm^−2^, and 60% potassium sulfate 150.00 kg hm^−2^); B1 (600.00 kg hm^−2^ of biochar-based fertilizer 150.00ion); BCK (600.00 maize the SPAD value at the filling stage. size of differenc kg hm^−2^); B3 (biochar-based fertilizer reduced by 10%, 540.00 kg hm^−2^); B4 (biochar-based fertilizer reduced by 15%, 510.00 kg hm^−2^); and B5 (biochar-based fertilizer reduced by 20%, 480.00 kg hm^−2^).

### 3.2 Effects of biochar-based fertilizer on soil nutrient content

Table 2 shows the effects of biochar-based fertilizer on soil nutrient contents at the growth stages of maize. Biochar fertilizer had no obvious effect on soil pH value at the silking and filling stages. Biochar-based fertilizer treatment had no obvious effect on the organic matter content of maize at the filling and maturity stages. The alkali-hydrolyzable N content in treatment B1 at the maturity stage showed an overall decreasing trend compared with BCK. The available P in the soil at silking, filling, and maturity stages of B1, increased by 0.93, 5.76, and 1.23%, compared with that of BCK. Compared with BCK, the available K content of B1 at silking and filling stages increased.

**Table 2:** Effect of biochar-based fertilizer on soil nutrient content at maize critical growth stages

Growth stage	Treatment	pH	The organic matter (g kg^−1^)	Alkaline hydrolysis N (mg kg^−1^)	Available P (mg kg^−1^)	Available K (mg kg^−1^)
Silking stage	BKB	5.54 ± 0.30^a^	31.16 ± 2.11^a^	180.00 ± 4.21^c^	32.60 ± 8.72^c^	84.00 ± 7.61^c^
	BCK	5.13 ± 0.21^ab^	31.31 ± 1.41^a^	207.00 ± 5.00^a^	53.50 ± 2.12^a^	97.00 ± 3.20^b^
	B1	4.91 ± 0.42^b^	32.44 ± 2.83^a^	200.00 ± 5.66^a^	54.00 ± 4.24^a^	117.00 ± 11.28^a^
	B2	5.17 ± 0.37^ab^	34.30 ± 2.53^a^	192.00 ± 3.83^b^	49.20 ± 2.61^a^	89.00 ± 3.00^c^
	B3	5.06 ± 0.26^ab^	32.32 ± 1.63^a^	192.00 ± 6.60^b^	42.10 ± 3.19^b^	119.00 ± 4.24^a^
	B4	4.82 ± 0.28^b^	34.44 ± 1.65^a^	192.00 ± 8.80^b^	42.30 ± 2.45^b^	97.00 ± 4.69^b^
	B5	5.10 ± 0.38^ab^	34.93 ± 2.05^a^	188.00 ± 8.50^b^	42.40 ± 5.30^b^	89.00 ± 8.20^c^
Filling stage	BKB	5.36 ± 0.27^a^	41.20 ± 3.02^a^	188.00 ± 8.29^a^	44.90 ± 2.37^a^	84.00 ± 3.42^a^
	BCK	5.18 ± 0.25^a^	35.50 ± 1.93^bc^	172.00 ± 3.35^b^	43.40 ± 3.16^abc^	87.00 ± 2.51^a^
	B1	5.13 ± 0.30^a^	33.30 ± 2.00^c^	172.00 ± 2.83^b^	45.90 ± 2.71^a^	89.00 ± 2.87^a^
	B2	5.01 ± 0.32^a^	36.20 ± 2.03^bc^	184.00 ± 8.55^a^	44.50 ± 2.75^ab^	89.00 ± 2.26^a^
	B3	5.30 ± 0.29^a^	34.40 ± 2.70^bc^	168.00 ± 9.63^b^	38.20 ± 3.04^c^	84.00 ± 2.68^a^
	B4	5.11 ± 0.27^a^	37.70 ± 1.69^ab^	184.00 ± 8.91^a^	39.10 ± 4.85^bc^	84.00 ± 4.16^a^
	B5	5.12 ± 0.42^a^	35.60 ± 2.40^bc^	172.00 ± 8.64^b^	38.10 ± 4.56^c^	87.00 ± 3.89^a^
Maturity stage	BKB	5.47 ± 0.24^a^	33.20 ± 1.64^a^	164.00 ± 4.56^ab^	37.30 ± 7.40^b^	89.00 ± 3.05^a^
	BCK	5.31 ± 0.21^ab^	33.00 ± 2.28^a^	168.00 ± 4.20^a^	48.60 ± 2.64^a^	91.00 ± 2.51^a^
	B1	4.91 ± 0.25^ab^	30.30 ± 2.49^a^	164.00 ± 4.56^ab^	49.20 ± 3.04^a^	91.00 ± 2.87^a^
	B2	5.01 ± 0.21^ab^	33.90 ± 2.65^a^	168.00 ± 4.18^a^	46.70 ± 2.18^a^	89.00 ± 2.34^a^
	B3	4.86 ± 0.31^b^	33.90 ± 2.83^a^	160.00 ± 4.75^bc^	46.00 ± 3.02^a^	89.00 ± 2.82^a^
	B4	5.24 ± 0.38^ab^	33.00 ± 1.50^a^	153.00 ± 6.70^c^	44.90 ± 2.37^a^	89.00 ± 2.51^a^
	B5	5.30 ± 0.36^ab^	33.00 ± 1.79^a^	153.00 ± 6.88^c^	45.90 ± 2.66^a^	89.00 ± 2.70^a^

Note: Letters a–c in the same column indicate significant difference at *p* < 0.05 (*n* = 3, LSD test).

### 3.3 Effects of biochar-based fertilizer on dry matter accumulation of maize

Table 3 shows the dry matter weight of each part of maize at the growth stages. In the silking stage, the aboveground dry matter weights of B1, B2, and B4 increased by 1.60, 1.16, and 5.98%, respectively, compared with BCK. In the filling stage, treatments B1 and B5 increased the dry matter weight of maize aboveground. As a result, the aboveground dry matter weight of B1 was higher than that of BCK. In the mature stage of maize, the dry matter weights of B1–B5 showed a decrease compared with that of BCK. However, the differences were not significant.

**Table 3:** Effect of biochar-based fertilizer on dry matter accumulation at maize critical growth stages

Growth stage	Treatment	Leaf weight	Sheath weight	Stem weight	Ear weight	Dry matter weight above ground
		(g/plant)	(g/plant)	(g/plant)	(g/plant)	(g/plant)
Silking stage	BKB	34.66 ± 0.82^b^	17.09 ± 0.53^a^	41.54 ± 1.73^a^	4.14 ± 1.00^b^	97.43 ± 0.76^a^
	BCK	36.49 ± 0.67^ab^	18.09 ± 0.53^a^	38.15 ± 1.17^a^	6.11 ± 0.98^ab^	98.83 ± 1.10^a^
	B1	38.08 ± 0.09^a^	16.97 ± 0.32^a^	40.39 ± 0.19^a^	4.97 ± 0.97^ab^	100.41 ± 0.84^a^
	B2	38.20 ± 1.44^a^	17.64 ± 0.52^a^	40.19 ± 4.15^a^	3.95 ± 0.53^b^	99.98 ± 5.29^a^
	B3	35.69 ± 0.81^ab^	17.27 ± 0.75^a^	38.59 ± 2.07^a^	4.66 ± 1.29^b^	96.21 ± 4.45^a^
	B4	36.96 ± 2.01^ab^	17.79 ± 0.74^a^	43.00 ± 2.56^a^	7.00 ± 1.65^a^	104.74 ± 6.66^a^
	B5	34.66 ± 2.31^b^	17.09 ± 1.11^a^	41.54 ± 2.77^a^	4.14 ± 1.96^b^	97.43 ± 7.92^a^
Filling stage	BKB	32.71 ± 5.12^b^	15.31 ± 1.82^b^	47.29 ± 4.70^bc^	95.00 ± 16.75^b^	190.30 ± 27.70^b^
	BCK	42.40 ± 1.65^a^	16.90 ± 1.57^a^	55.31 ± 1.88^abc^	127.50 ± 8.16^a^	242.11 ± 12.25^a^
	B1	41.99 ± 1.64^a^	19.58 ± 0.86^ab^	53.44 ± 2.65^ab^	127.50 ± 7.59^a^	242.51 ± 11.19^a^
	B2	42.36 ± 2.42^a^	20.15 ± 0.92^a^	55.42 ± 2.38^ab^	117.50 ± 6.61^a^	235.43 ± 12.12^a^
	B3	39.00 ± 3.30^ab^	19.34 ± 1.64^ab^	51.95 ± 2.66^bc^	112.50 ± 2.39^ab^	222.79 ± 7.82^ab^
	B4	38.10 ± 3.02^ab^	19.69 ± 1.65^a^	56.97 ± 2.21^ab^	110.00 ± 8.54^ab^	224.76 ± 14.69^a^
	B5	41.63 ± 1.33^a^	20.10 ± 1.14^a^	59.96 ± 1.71^a^	125.00 ± 6.45^a^	246.68 ± 9.27^a^
Maturity stage	BKB	31.95 ± 8.49^a^	11.73 ± 3.13^b^	41.97 ± 7.12^ab^	276.88 ± 47.03^a^	362.53 ± 58.66^a^
	BCK	31.65 ± 3.24^a^	15.19 ± 1.98^ab^	45.50 ± 5.47^ab^	308.75 ± 63.24^a^	401.09 ± 71.80^a^
	B1	29.07 ± 9.00^a^	15.50 ± 3.09^ab^	45.44 ± 9.18^ab^	300.63 ± 59.29^a^	390.63^a^ ± 72.59
	B2	31.55 ± 3.94^a^	15.90 ± 4.46^ab^	42.73 ± 4.85^ab^	300.63 ± 30.94^a^	390.80 ± 40.23^a^
	B3	28.36 ± 3.35^a^	13.10 ± 1.83^ab^	38.23 ± 3.93^b^	265.00 ± 26.90^a^	344.70 ± 30.32^a^
	B4	33.73 ± 6.62^a^	15.32 ± 3.22^ab^	43.84 ± 3.99^ab^	308.13 ± 27.06^a^	401.01 ± 35.86^a^
	B5	34.61 ± 5.36^a^	16.95 ± 2.75^a^	50.36 ± 8.41^a^	318.13 ± 56.79^a^	420.04 ± 69.61^a^

Note: Letters a–c in the same column indicate significant difference at *p* < 0.05 (*n* = 10, LSD test).

These results indicated that treatment B1 could increase the dry matter weight of maize shoot at silking and filling stages. This may be because biochar-based fertilizers delay nutrient release in soil. Consequently, the amount of nutrients released is basically consistent with the nutrient requirement of maize; thus, it promotes the accumulation of dry matter.

### 3.4 Effects of biochar-based fertilizer on the nutrient content of maize plants

**Figure 1:**
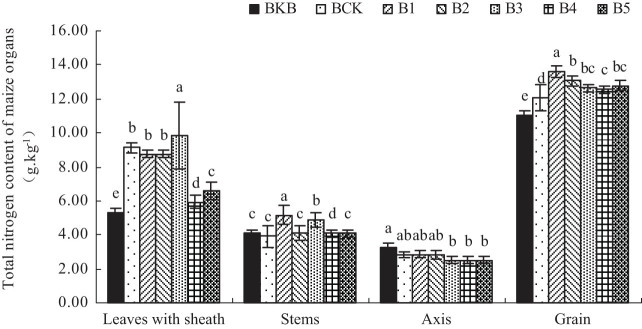
Effect of biochar-based fertilizer on total N content in maize organs. Note: Different letters indicate significant difference of treatments at *p* < 0.05 (*n* = 3, LSD test).

Figure 1 shows the total N content in each organ of the maize plant. Biochar-based fertilizer treatments B1, B2, B4, and B5 reduced the total N content of maize leaves and sheaths. B1–B5 treatments increased the total N content of maize stem compared with BCK’s 3.90 g kg^−1^. Treatments B1 and B2 increased the total N content of the maize axis at 13.60 and 13.07 g kg^−1^. B1–B5 increased the total N content of maize grains compared with BCK’s 12.08 g kg^−1^.

**Figure 2:**
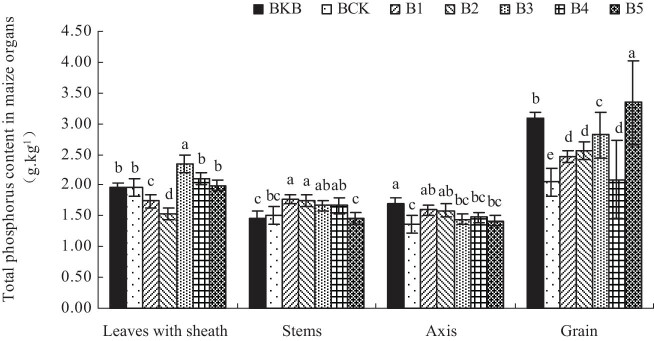
Effect of biochar-based fertilizer on total P content in maize organs. Note: Different letters indicate significant difference of treatments at *p* < 0.05 (*n* = 3, LSD test).

Figure 2 shows the total P content in each maize organ. Treatments B1–B4 increased the total P content in maize stems compared with 1.51 g kg^−1^ in BCK. Treatments B1–B5 increased the total P content of the maize axis. B1–B5 increased the total P content of maize grains, and B1–B5 increased by 20, 24.88, 37.56, 1.95, and 62.93% compared with BCK, respectively.

**Figure 3:**
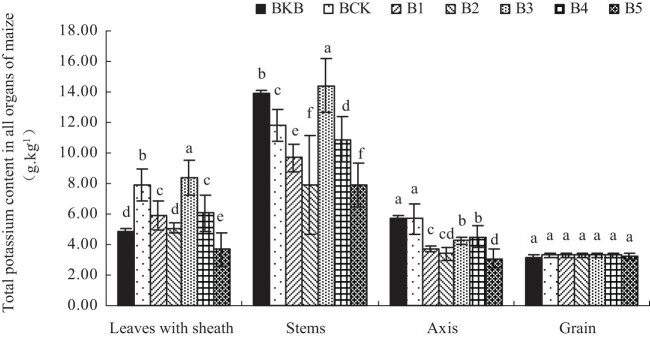
Effect of biochar-based fertilizer on total K content in maize organs. Note: Different letters indicate significant difference of treatments at *p* < 0.05 (*n* = 3, LSD test).

Figure 3 shows the total K content in each organ of the maize plant. It can be seen that treatments B1, B2, B4, and B5 all reduced the total K content of maize organs.

**Figure 4:**
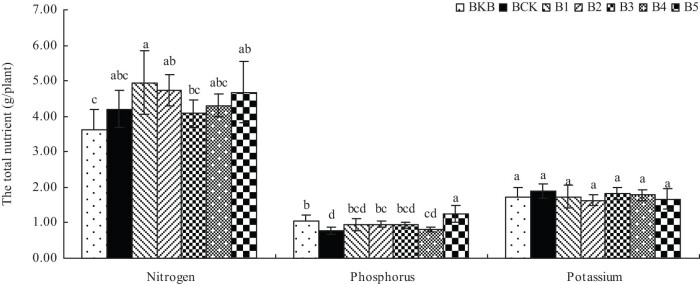
Effects of biochar-based fertilizer on total N, P, and K content of maize plants. Note: Different letters indicate significant difference of treatments at *p* < 0.05 (*n* = 3, LSD test).

As shown in Figure 4, each treatment of biochar-based fertilizer increased the total phosphorus content of maize plants, and treatments B1, B2, B4, and B5 increased the total nitrogen content of maize plants. However, each treatment of biochar-based fertilizer reduced the total K content of maize plants.

### 3.5 Effects of biochar-based fertilizer on the nutrient utilization rate of maize

Table 4 shows the effects of biochar-based fertilizer on the nutrient utilization rate of maize. Biochar-based fertilizer treatment increased the agronomic efficiency of fertilizer on the whole (except B3 treatment). The total partial fertilizer productivity of B1–B5 increased by 39.64, 31.57, 35.28, 43.91, and 54.57%, respectively, compared with that of BCK. This indicates that conventional fertilizer treatment (BCK) of maize has certain drawbacks; a large number of fertilizers not only failed to achieve a significant yield increase but also caused fertilizer waste, greatly reducing the fertilizer utilization rate, but biochar-based fertilizer is more conducive to the absorption and utilization of nutrients for maize than conventional fertilizer.

**Table 4:** Effects of biochar-based fertilizer on nutrient utilization rate of maize

Treatment	Fertilizer agronomic efficiency (kg kg^−1^)	Fertilizer partial productivity (kg kg^−1^)
BCK	5.00 ± 1.40^b^	33.60 ± 1.40^e^
B1	10.36 ± 0.26^a^	46.92 ± 0.26^bc^
B2	5.72 ± 0.20^b^	44.21 ± 0.20^d^
B3	4.83 ± 0.00^b^	45.25 ± 0.00 ^cd^
B4	5.34 ± 1.88^b^	48.35 ± 1.88^b^
B5	6.23 ± 1.28^b^	51.94 ± 1.28^a^

Note: Letters a–d in the same column indicate significant difference at *p* < 0.05 (*n* = 3, LSD test).

### 3.6 Effects of biochar-based fertilizer on maize agronomic traits

Table 5 shows the effects of biochar-based fertilizer on maize agronomic traits. The ear length of B1 and B3 increased by 5.43 and 1.23% compared with BCK. The ear diameter of maize under B1, B3, and B4 treatments increased by 2.47, 1.2, and 0.82% compared with BCK. The stem diameter of B1 was 2.09% higher than that of BCK. B1, B3, and B4 treatments increased the plant height of maize compared with BCK, respectively.

**Table 5:** Effects of biochar-based fertilizer on agronomic traits of maize

	Ear length (cm)	Ear coarseness (cm)	Thick stems (cm)	Plant height (cm)
BKB	20.50 ± 1.03^ab^	4.79 ± 0.20^a^	1.64 ± 0.13^b^	255.50 ± 4.38^a^
BCK	20.25 ± 1.09^ab^	4.85 ± 0.08^a^	1.91 ± 0.17^ab^	261.50 ± 6.69^a^
B1	21.35 ± 1.25^a^	4.97 ± 0.22^a^	1.95 ± 0.20^a^	264.50 ± 13.22^a^
B2	19.15 ± 1.68^b^	4.85 ± 0.12^a^	1.76 ± 0.22^ab^	254.00 ± 8.10^a^
B3	20.50 ± 1.51^ab^	4.91 ± 0.17^a^	1.86 ± 0.25^ab^	266.00 ± 9.37^a^
B4	19.95 ± 0.96^ab^	4.89 ± 0.13^a^	1.88 ± 0.16^ab^	265.50 ± 8.32^a^
B5	19.95 ± 1.88^ab^	4.85 ± 0.22^a^	1.87 ± 0.23^ab^	258.50 ± 19.73^a^

Note: Letters a–b in the same column indicate significant difference at *p* < 0.05 (*n* = 10, LSD test).

### 3.7 Effects of biochar-based fertilizer on maize yield

**Figure 5:**
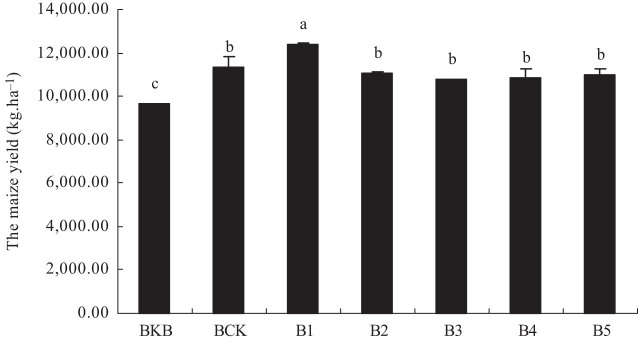
Effect of biochar-based fertilizer on maize yield. Note: Different letters indicate significant difference of treatments at *p* < 0.05 (*n* = 3, LSD test).

Figure 5 shows the effect of biochar-based fertilizer on maize yield. The maize yield of B1 was 12386.25 kg hm^−2^, which was 9.23% higher than that of BCK (11340.00 kg hm^−2^), and the difference was significant (*p* < 0.05). The maize yields of B2, B3, B4, and B5 were 2.23, 4.76, 4.32, and 3.27% lower than those of BCK (11340.00 kg hm^−2^), but the difference was not significant. These results indicated that under the same quality application of biochar-based fertilizer as BCK, maize yield could be increased by using standard ridge mode (ridge spacing 65 cm). Under the condition of 5–20% reduction of biochar-based fertilizer application, maize could be in a stable yield level.

### 3.8 Correlation analysis of maize yield with soil nutrient content and dry matter accumulation

Table 6 shows the correlation analysis table of maize yield, soil nutrient content, and dry matter accumulation. The yield of maize was positively correlated with total N content and positively correlated with organic matter content at the jointing stage. There was a significant positive correlation between yield and alkali-hydrolyzed N content at the jointing stage. The yield was positively correlated with the available P and available K content at different stages.

**Table 6:** Correlation analysis of maize yield with soil nutrient content and dry matter accumulation

Growth stage	Parameters	Yield	Ear length	Ear coarse	Stem stems	Plant height
Mature stage	Plant total N	0.84**	−0.04	0.61	0.61	0.08
	Plant total P	−0.26	−0.07	−0.28	−0.30	−0.46
	Plant total K	0.07	0.40	0.17	0.39	0.64
	Dry matter accumulation	0.39	−0.30	−0.01	0.42	−0.14
Jointing stage	SPAD value	0.38	0.40	0.54	0.19	0.40
Filling stage	SPAD value	0.03	0.14	0.09	0.06	0.34
Jointing stage	pH	−0.71*	−0.11	−0.81*	−0.86**	−0.75*
	Organic matter	0.15	−0.59	0.12	0.19	−0.08
	Hydrolyzable N	0.78*	0.2	0.52	0.78*	0.45
	Available P	0.91**	0.1	0.58	0.73*	0.25
	Available K	0.6	0.63	0.88**	0.65	0.81*
Filling stage	pH	−0.58	0.51	−0.27	−0.4	0.13
	Organic matter	−0.87**	−0.26	−0.80*	−0.84**	−0.51
	Hydrolyzable N	−0.57	−0.42	−0.57	−0.74*	−0.56
	Available P	0.21	0.24	−0.04	−0.26	−0.41
	Available K	0.73*	−0.07	0.3	0.33	−0.3
The mature stage	pH	−0.61	−0.19	−0.80*	−0.45	−0.44
	Organic matter	−0.70	−0.75*	−0.64	−0.51	−0.35
	Hydrolyzable N	0.15	0.04	−0.12	−0.23	−0.39
	Available P	0.91**	0.06	0.7	0.87**	0.43
	Available K	0.72*	0.57	0.44	0.59	0.31

Note: **, significantly different at 0.01 level; *, significantly different at 0.05 level.

## 4 Discussion

### 4.1 Effects of biochar-based fertilizer on the SPAD value of maize and soil nutrient content

Chlorophyll is the basic substance for photosynthesis in green plants and the main photosynthetic pigment of crop leaves. It affects the photosynthetic performance of crops, and its content reflects the senescence degree of leaves to a certain extent [20]. All treatments reduced the SPAD value at the filling stage, possibly because biochar-based fertilizer reduced the available N content in the soil at the mature stage of maize (Table 4) and the total N content in the leaves at the mature stage (Figure 1).

The results of Gao’s study showed that the biochar-based fertilizer increased soil pH value by 7.2% compared with NPK fertilizer treatment [21]. Yang et al. (2015) showed that the application of biochar-based fertilizer had no significant effect on soil pH value after three consecutive years [22]. In the mature stage of maize, biochar-based fertilizer reduces soil pH value. This may be due to the increase of maize root growth in different fertilization treatments. The massive growth of roots leads to an increase in the secretion of organic acids, which leads to a decrease in soil pH [23].

Yang et al. (2015) showed that biochar-based fertilizer for three consecutive years could increase SOM content [22]. Biochar-based fertilizer increased the organic matter content of albic soil in the silking stage of maize. This may be because the biochar input inhibits the mineralization of SOM and promotes the process of soil humification, leading to the increase of SOM content [24].

The results of Gao’s study showed that the application of biochar-based fertilizer was 2.1% lower than NPK treatment [21]. Wang (2020) found that biochar-based fertilizer can increase the content of soil available N [25]. The alkali-hydrolyzable N content of albic soil at maize maturity stage was decreased by biochar-based fertilizer compared with BCK. This is because under the condition of biochar reduction fertilization, the amount of N input to soil gradually decreased, and then reduced the content of alkali-hydrolyzed N. Biochar-based fertilizer may increase the ratio of C to N in soil [26], thus reducing the rate of soil microbial mineralization of soil organic N. After biochar-based fertilizer was applied to the soil, it disturbed the surface soil greatly, changed the pore structure of the soil, increased the aeration performance of the soil, and may accelerate the volatilization of NH_3_ in the fertilizer. Alternatively, the application of biochar-based fertilizer may promote the absorption and utilization of alkali-hydrolyzed N in maize.

Application of biochar-based fertilizer increased soil available P content compared with no fertilization [21]. Biochar-based fertilizer B1 increased the content of available soil P of albic soil. This may be because biochar enhances the adsorption of phosphate and soluble organophosphorus, effectively reduces the absorption of iron oxide on P, and reduces the leaching loss of available P [24]. Biochar, meanwhile, is itself an important source of P [27].

Previous studies have found that the content of available K in biochar-based fertilizer decreased by 1.3% compared with NPK treatment [21]. Biochar-based fertilizer B1 treatment is beneficial for increasing maize’s available K content. This is because biochar itself contains a large amount of K, which directly increases the content of available K in the soil after application. At the same time, biochar reduced K leaching. Biochar has a special pore structure that slows down the infiltration rate of water and enhances the cation exchange capacity of the soil, thus improving the adsorption capacity of soil for K^+^ in solution with strong mobility and easy leaching and reducing the leaching of K [21]. Biochar may enter the soil mineral layer and react and compete with fixed K ions, so that part of the inactive K can be converted into available K [28].

### 4.2 Effects of biochar-based fertilizer on the nutrient content of maize plants

Previous studies have found that biochar-based fertilizer can increase the N, P, and K contents of rice plants [11,29,30]. Biochar-based fertilizer treatments B1–B5 increase the total nitrogen and total phosphorus content of maize grains. Treatments B1–B2 and B4–B5 increased the total nitrogen content of maize plants, and treatments B1–B5 increased the total phosphorus content of maize plants. This is because the biochar-based fertilizer can promote the uptake and utilization of N and P nutrients in maize, which may be because biochar has a large amount of nutrient content and can improve soil fertility. Biochar has a large specific surface area, rich in pore structure and surface negative charge, which can effectively reduce the infiltration rate of water and strengthen the adsorption capacity of nutrient elements [31,32]. Biochar can promote the activities of soil microorganisms, enhance the activities of various soil enzymes, and improve soil nutrient cycling [33,34,35,36,37], thus promoting the absorption of soil N, P, and other nutrients by maize. However, each treatment of biochar-based fertilizer reduced the total K content of maize plants because the K content of biochar-based fertilizer was lower than that of BCK, which led to the decrease in the total K content of maize plants treated with biochar-based fertilizer.

### 4.3 Effects of biochar-based fertilizer on maize yield

The growth of maize is a process of dry matter accumulation. The dry matter quality expresses the accumulation of maize assimilation, and the accumulation of total dry matter determines yield to a certain extent [38]. Previous studies revealed that biochar-based fertilizer could increase dry matter accumulation at the maize seedling stage [39]. The biochar-based fertilizer could increase the agricultural utilization rate and partial productivity of N, P, and K fertilizer in rice [40]. Biochar-based fertilizer treatment B1 increases maize yield. The maize yield of B2, B3, B4, and B5 shows no significant difference compared with that of BCK, and fertilizer input was saved 5–20% by biochar-based fertilizer. That is because biochar carries many nutrients on its own. Biochar is usually rich in N, P, K, Ca, and Mg, effectively improving soil fertility levels, and is an important material basis for promoting maize yield increase [38]. Meanwhile, biochar has special physical and chemical properties. Biochar has a great specific surface area, pore structure, and high CEC. It can help enhance the soil’s ability to intercept nutrients and be suitable for the growth of soil microbial breeding habitats, improve the metabolic activities of microorganisms, and promote the soil nutrient cycle to increase yield [41,42]. By observing the growth trend of maize at the maturity stage, the author found that the application of biochar-based slow-release fertilizer (B1) could reduce the occurrence of premature senescence and maintain the green leaf area for a long time, which may also be an important reason to promote the increase of maize yield.

### 4.4 Application prospect of biochar-based fertilizer

In the past, excessive application of nitrogen and phosphorus fertilizers has caused a serious nutrient loss, resulting in a large amount of nitrogen and phosphorus into water, causing agricultural nonpoint source pollution [1]. Due to the high stability and strong adsorption performance of biochar, the coating material of conventional nitrogen and phosphorus fertilizer of biochar crops can be used to produce biochar-based fertilizer to realize low-carbon agriculture and nitrogen and phosphorus co-emission reduction and reduce fertilizer application amount [10]. Therefore, it is necessary to develop special biochar-based fertilizers with different nutrient release characteristics for different crop types, soil types, and climate conditions to meet the dual requirements of crop growth and agricultural environmental protection in different regions.

## 5 Conclusion

Biochar-based fertilizer increased the total P of maize plants and the fertilizer’s partial productivity. Treatment B1 increased the dry-matter weight of the maize at silking and filling stages. Compared with BCK, the yield of B1 increased by 9.23%. Biochar-based fertilizer treatments (B2–B5) reduced maize yield, but there was no significant difference between their yield and BCK. This study aimed to provide a basic understanding and reference for maize fertilizer reduction with good application prospects.
